# Topographical Distribution and Spatial Interactions of Innate and Semi-Innate Immune Cells in Pancreatic and Other Periampullary Adenocarcinoma

**DOI:** 10.3389/fimmu.2020.558169

**Published:** 2020-09-10

**Authors:** Sebastian Lundgren, Patrick Micke, Jacob Elebro, Margareta Heby, Ina Hrynchyk, Björn Nodin, Karin Leandersson, Artur Mezheyeuski, Karin Jirström

**Affiliations:** ^1^Department of Clinical Sciences Lund, Oncology and Therapeutic Pathology, Lund University, Lund, Sweden; ^2^Department of Immunology, Genetics and Pathology, Uppsala University, Uppsala, Sweden; ^3^City Clinical Pathologoanatomic Bureau, Minsk, Belarus; ^4^Department of Translational Medicine, Division of Cancer Immunology, Lund University, Lund, Sweden

**Keywords:** dendritic cells, innate immunity, natural killer cells, natural killer T-cells, tumor microenvironment

## Abstract

**Background:**

The clinical management of pancreatic and other periampullary neoplasms remains challenging. In contrast to other cancer types, immunotherapies are largely ineffective, and the reason for the deprived immune response and the immune inhibiting cellular composition is only fragmentarily understood. The aim of this study was to comprehensively map the abundance, topographic distribution and spatial interaction of innate and innate-like immune cells in the tumor microenvironment of periampullary adenocarcinoma.

**Methods:**

Multiplexed immunofluorescent imaging was performed on tissue microarrays with tumors from a consecutive cohort of 175 patients with resected periampullary adenocarcinoma. To obtain a detailed spatial analysis of immune cell infiltration, two multiplex immune panels including antibodies against CD3, NKp46, CD56, CD68, CD163 and CD1a, CD208, CD123, CD15, CD68 and pan-cytokeratin were applied.

**Results:**

The infiltration of natural killer (NK) and NK-like T (NKT) cells was lower in malignant compared to benign tissue. NKT cells were more abundant in intestinal type compared to pancreatobiliary type tumors, and were associated with more favorable clinicopathological features and a prolonged survival. The interaction of NKp46^+^ NKT cells with macrophages was also associated with a prolonged survival.

**Conclusions:**

This study provides a comprehensive map of the innate immune landscape in periampullary adenocarcinoma. NK cells, and even more so NKT cells, are revealed to be central players in the local immune response in a clinically relevant context.

## Introduction

Periampullary adenocarcinomas encompass cancers arising in the area around the ampulla of Vater, i.e., in the distal bile duct, the pancreatic head, the duodenum or the ampulla. There is increasing evidence that tumor morphology, i.e., classification into intestinal type (I-type) or pancreatobiliary type (PB-type), is a more important determinant of patient outcome than tumor origin (1–4). Despite recent therapeutic advances, the overall survival rates for patients with periampullary adenocarcinoma remain as low as around 5% (5), and pancreatic cancer is the fourth leading cause of cancer related death in Europe (6). Although targeted therapies and immunotherapy are standard of care for many advanced solid tumors today, early clinical studies on these types of therapies in periampullary and pancreatic cancer have been disappointing (7).

Adding to this, the mutational burden of pancreatic cancer is low, leading to a low antigenicity that hinders an efficient endogenous immune response. Mutational burden has emerged as a promising predictive biomarker for response to immunotherapy, which may partly explain why early trials of immunotherapy in pancreatic cancer have failed to show any treatment benefit. There is, however, increasing evidence that a high mutational burden does not always correspond to an immunologically “hot” tumor and, hence, an effective anti-tumoral immune response (8). Therefore, in order to improve patient outcome, there is a great need to further decipher the biological basis underlying the nearly universal treatment resistance of these tumors, and to identify not only novel treatments, but also improved strategies for treatment stratification and appropriate complementary biomarkers.

Most previous studies on the immune microenvironment of pancreatic and other periampullary cancers have relied on single markers, not taking into account the full complexity of leukocyte subpopulations. Multiplex immunofluorescence approaches have been shown to more accurately predict response to immunotherapy compared to standard immunohistochemistry, tumor mutational burden and gene expression (9). Recently, close interaction of immune cells with cancer cells was demonstrated to predict response to checkpoint blockade in melanoma (10), highlighting the importance of also taking the spatial interactions of immune cells within the tumor microenvironment into account. Additionally, the topography of leukocyte infiltration is seldom considered (11). Thus, a more comprehensive analysis of the phenotypic composition of tumor-infiltrating leukocytes in periampullary and pancreatic cancer may provide new insights into how different tumors shape their respective immune landscape.

We have previously shown that the composition and spatial interaction of various subsets of T and B lymphocytes in the tumor microenvironment of resected periampullary adenocarcinoma affects clinical outcome, not least depending on morphological type (12). In this study, we set forth to investigate the composition of the innate immune landscape in these tumors, using high resolution multiplex immunofluorescence.

## Materials and Methods

### Study Cohort

The study cohort consists of a retrospective, consecutive series of primary tumors from all 175 patients with periampullary adenocarcinoma who underwent pancreaticoduodenectomy in the University hospitals of Malmö and Lund from January 1st 2001 to December 31 2011 (13, 14). Follow-up started at the date of surgery and ended at death or on March 31 2017. Data on vital status were obtained from the Swedish National Civil Register. Clinical data including information on adjuvant treatment were obtained from patient charts. All 175 cases underwent strict histopathological re-evaluation before the start of the study (13). Sixty-five tumors were classified as I-type, and 110 as PB-type, with their anatomical origin being 14 duodenal, 70 ampullary, 45 distal bile duct and 46 pancreatic. Paired benign tissue was available from 34 cases, nine of which were I-type tumors and 25 PB-type tumors. Mismatch repair (MMR) deficiency was assessed as previously described (15).

### Targeted Next Generation DNA Sequencing

Next generation DNA sequencing was performed as previously described (4). In brief, the mutational status of 70 commonly cancer-associated genes was assessed using the Illumina TruSeq custom amplicon assay (Illumina, San Diego, United States) according to the instructions obtained from the manufacturer. Variant calling and annotation, quality filtering and alignment were performed using the standard analysis pipeline (Illumina, San Diego, United States). Detected mutations were screened against ExAC and COSMIC databases in order to filter out common single nucleotide polymorphisms.

### Tissue Microarray Construction and Multiplex Immunofluorescent Staining

Tissue microarrays (TMA) were constructed as previously described (16, 17). In brief, a semi-automated arraying device (TMArrayer, Pathology Devices, Westminster, United States) was used to obtain a set of three one-millimeter cores from viable, non-necrotic areas of the primary tumors. Panel 1 consisted of antibodies against CD68 (M0876, Dako; Agilent Technologies, Santa Clara, CA, United States, diluted 1:100), CD163 (HPA046404, Atlas Antibodies, Bromma Sweden, 1:100), NKp46 (PA5-79720, Thermo Fisher Scientific, Rockford, IL, United States, 1:150), CD56 (M730429-2, Dako; Agilent Technologies, 1:100) and CD3 (M725429-2, Dako; Agilent Technologies, 1:80). Panel 2 consisted of antibodies against CD1a (M357101-2, Dako; Agilent Technologies, 1:400), CD208 (PA5-84069, Thermo Fisher Scientific 1:50), CD123 (198M-14, Sigma, Saint Louis, United States, 1:20), CD15 (M3631, Sigma, 1:100) and CD68 (M0876, Dako; Agilent Technologies, 1:800).

In both panels, pan-cytokeratin staining was carried out with a combination of the following antibodies: anti-E-cadherin (610182, BD Biosciences, Franklin Lakes, NJ, United States, 1:2000), anti-pan Cytokeratin (ab7753, Abcam, San Francisco, CA, United States, 1:1000) and anti-pan Cytokeratin (MA5-13156, Thermo Fisher Scientific, 1:500). The TMAs were stained with 4’,6-diamidino-2-phenylindole (Spectral DAPI, Akoya Bioscience, San Francisco, CA, United States) in order to visualize cell nuclei. Lymphocyte infiltration was analyzed as previously described (12).

### Tissue Microarray Imaging, Analysis and Thresholding

Tissue microarrays imaging was performed using the Vectra Polaris System (Akoya Bioscience) as previously described (18). TMA cores were scanned at ×10 magnification in order to annotate cores and select regions for multispectral imaging. Multispectral imaging was then performed at a resolution of 2 pixels per 1 μm. Spectral unmixing was performed using inForm software (Akoya Bioscience). Each TMA core was manually assessed by a board-certified pathologist in order to exclude non-viable areas such as non-tumor tissue, necrosis and staining artifacts. Subsequently, the image analysis was carried out in two steps; a training session and an image analysis session. In order to set up a machine learning algorithm for tissue segmentation, a set number of cores was categorized into three types of tissue compartments (tumor, stromal or blank areas). Cell segmentation was performed using DAPI. The perinuclear area was defined as the 3 μm (6 pixels) area surrounding the nuclei and thus considered to be cytoplasm. The total cell area (including cytoplasm and nuclei) was evaluated for cytoplasmic and membranous protein expression. Immune cell density was normalized against the total area of viable tissue. To define the intensity threshold for biomarker positivity, the inForm software used a random selection of TMA cores, and the defined thresholds were then applied to the entire cohort. Co-expression data were used to identify immune cell subtypes. Immune cell infiltration was assessed as the number of cells per compartment (tumor and stroma) as well as in the total viable area of each TMA core (referred to as total count). Spatiality of immune cells was assessed both in regard to cancer cells and to other immune cells, where the distances to the nearest cancer cell and neighboring immune cell was measured. The potential zone of interaction was defined to a radius of 15 μm based on previously published research (19).

### Data Processing and Statistical Analyses

Wilcoxon signed rank test was used for comparison of two related samples. Mann-Whitney U test was used to illustrate differences between categorical and continuous variables. Unsupervised hierarchal clustering was performed in order to identify immune cell signatures. For all survival analyses, cases were dichotomised using the median density of immune cell infiltration as a cut-off. Cox regression proportional hazards models were used to estimate hazard ratios (HR) for death within 5 years in both univariable and multivariable analyses. The multivariable model included adjustment for age (continuous), T-stage (T1–T2 vs. T3–T4), N-stage (negative vs. positive nodal status), grade (well-moderate vs. poor), adjuvant chemotherapy (none vs. any), invasion into vascular and lymphatic structures, and perineural growth. Morphology (I-type vs. PB-type) was included in the multivariable model in analyses of the entire cohort but not in the morphology-stratified analyses. Two patients with PB-type tumors who received neoadjuvant therapy were excluded from the statistical analyses. Three additional cases were excluded from the survival analyses; one patient with a PB-type tumor who was lost to follow-up due to emigration, and two patients with I-type tumors who died of complications within 1 month from surgery. All statistical calculations were performed with SPSS version 24.0 (SPSS Inc., Chicago, IL, United States) or with R software, version 3.6.0 (R Foundation for Statistical Computing, Vienna, Austria) with the integrated development environment RStudio, version 1.1.456 (RStudio Team, Boston, MA, United States). All statistical tests were two-sided and *p*-values <0.05 were considered significant.

## Results

### Definition of Immune Cell Subsets

**FIGURE 1:**
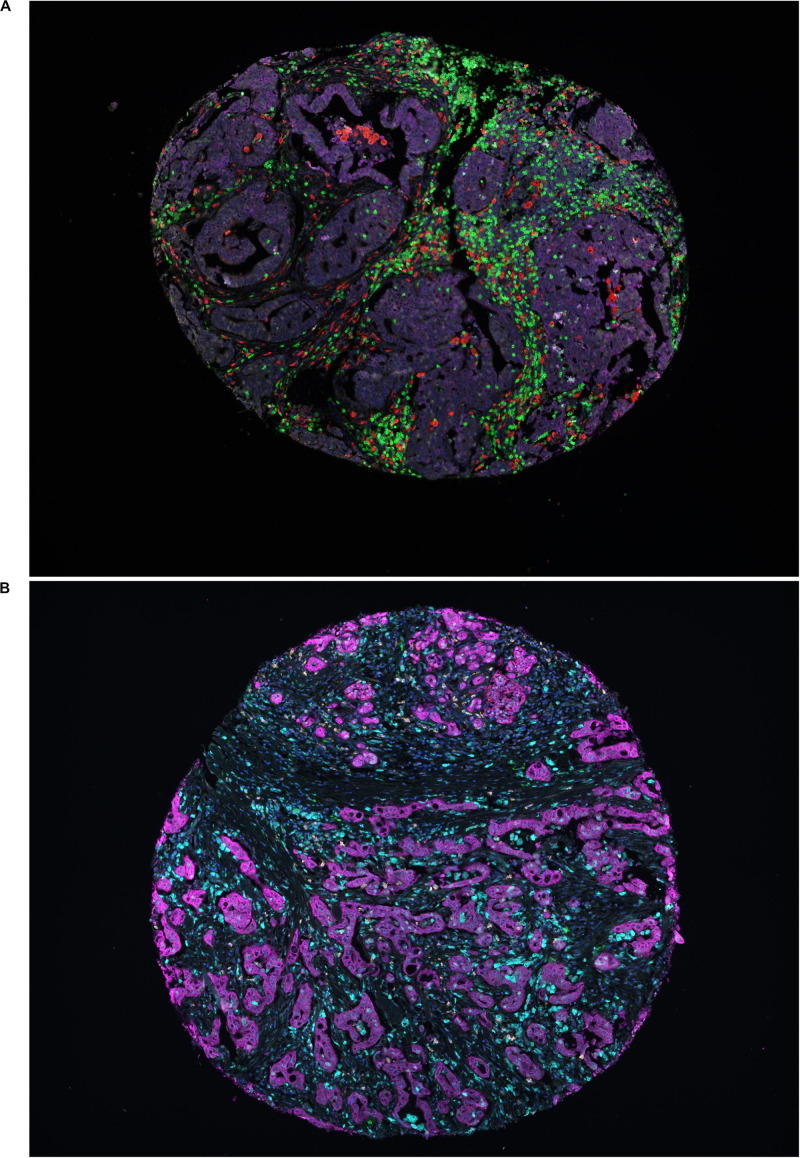
Representative immunofluorescence images. **(A)** Representative image of a TMA core with heterogeneous infiltration of leukocytes in panel 1; red denoting CD68, yellow denoting CD56, green denoting CD3, pink denoting NKp46, cyan denoting CD163 and magenta denoting cytokeratin. **(B)** Representative image of a TMA core with heterogeneous infiltration of leukocytes in panel 2; green denoting CD1a, red denoting CD208, yellow denoting CD15, pink denoting CD123, cyan denoting CD68 and magenta denoting cytokeratin.

Immune cell infiltration was possible to determine in 164 (94%) cases with panel 1 and in 160 (91%) cases with panel 2. Sample multiplex immunofluorescent staining images representing both panels are shown in Figure 1.

Combined marker expression levels in each cell were used in order to identify relevant immune cell subpopulations. In immune panel 1 the following cell types were defined: CD68^+^ macrophages (single-positive), CD163^+^ anti-inflammatory myeloid cells (single-positive), CD68^+^CD163^+^ macrophages, CD3^–^CD56^+^NKp46^+^ natural killer (NK) cells, CD3^+^CD56^+^ NKT cells (CD56^+^ NKT cells), CD3^+^NKp46^+^ NKT cells, and CD3^+^CD56^+^NKp46^+^ NKT cells. Hence, the defined NKT subpopulations described herein could partly consist of γδ T-cells (20). In immune panel 2, three dendritic cell (DC) single marker positive classes were characterized: CD1a^+^CD15^–^ immature DCs (iDC), CD208^+^CD15^–^ mature DCs (mDC), and CD123^+^CD15^–^ plasmacytoid DCs (pDC). Three granulocyte classes were identified by co-expression of CD15: CD1a^+^CD15^+^, CD208^+^CD15^+^ and CD123^+^CD15^+^ (myeloid progenitors or basophils). CD68 was used as an exclusion marker for macrophage populations in panel 2.

### Heterogeneity of Immune Cell Infiltration in the Tumor Microenvironment

Pairwise comparisons of immune cell infiltration in benign versus malignant tissue and in tumor versus stromal compartments, respectively, are shown in Additional File 1. Infiltration of NK cells, CD56^+^ NKT cells and CD56^+^NKp46^+^ NKT cells was significantly lower in malignant than in benign tissue. In contrast, infiltration of CD68^+^ macrophages, iDCs, pDCs and CD123^+^CD15^+^ granulocytes, the latter of which may be myeloid progenitors or more likely basophils, was significantly higher in malignant than in benign tissue.

Infiltration of NK cells, iDCs, mDCs and CD123^+^CD15^+^ granulocytes was significantly higher in the tumor than in the stromal compartment, whereas infiltration of all macrophage populations and CD56^+^ NKT and NKp46^+^ NKT cells was significantly higher in the stromal than in the tumor compartment.

### Immune Cell Density in Relation to Morphology and Anatomical Origin

**FIGURE 2:**
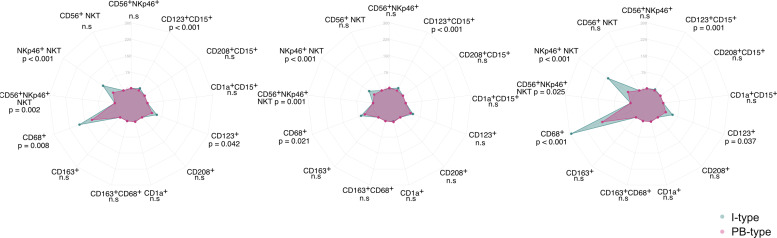
Associations of immune cell subsets with tumor morphology. Radar plots of leukocyte infiltration levels in PB-type tumours (purple) and I-type tumors (blue). Data shown separately for total tissue (left), the tumor compartment (middle) and the stromal compartment (right).

**FIGURE 3:**
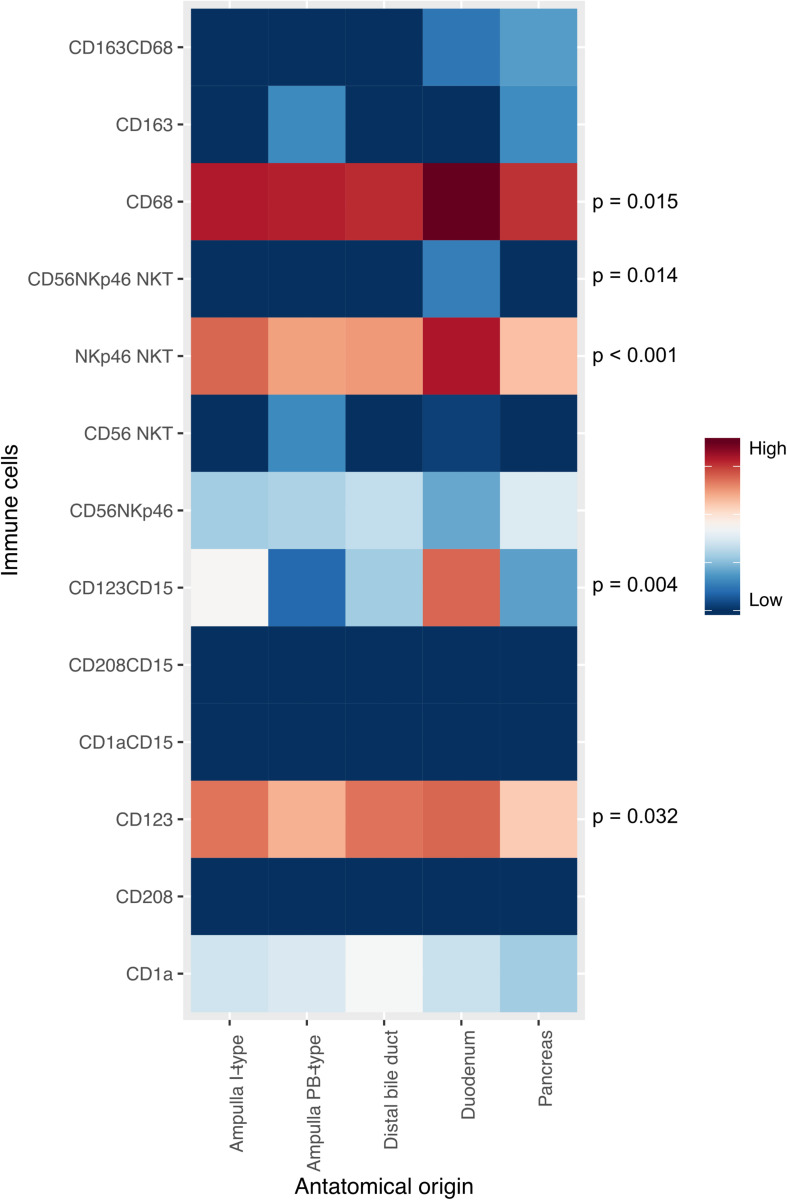
Associations of immune cell subsets with clinicopathological factors. Heatmap illustrating differences in densities of leukocyte subclass infiltration according to anatomical origin.

Radar charts and heatmaps depicting the relative proportion of immune cell infiltration according to tumor morphology and anatomical origin, respectively, are shown in Figures 2, 3.

Several immune cell populations; CD68^+^ macrophages, NKp46^+^ NKT cells, CD56^+^NKp46^+^ NKT cells and CD123^+^CD15^+^ granulocytes, had significantly higher infiltration levels in I-type tumors compared to PB-type tumors, both in the tumor and in the stromal compartments (Figure 2).

Tumors with duodenal origin had the highest infiltration levels of NKp46^+^ NKT cells, CD56^+^NKp46^+^ NKT cells, CD68^+^ macrophages and CD123^+^ and CD123^+^CD15^+^ granulocytes (Figure 3).

### Immune Cell Density in Relation to Clinicopathological Characteristics

The associations between different immune cells and clinicopathological characteristics are shown in Additional File 2. High infiltration of several immune cell populations, notably in particular NKT cells, was significantly associated with more favorable tumor characteristics such as absence of growth into blood vessels, lymphatic vessels, perineural structures and peripancreatic fat. Additionally, high infiltration of iDCs was significantly associated with poor differentiation grade and high infiltration of NKp46^+^ NKT cells was significantly associated with a more advanced T-stage. There were no significant associations between any of the immune cell populations and sex (data not shown).

### Immune Cell Density in Relation to Common Mutations and Mismatch Repair Status

The abundance of different immune cells in relation to the most common mutations (prevalence >10%) is shown in Additional File 3. *KRAS* mutated tumors had a significantly lower infiltration of NKp46^+^ NKT cells in the tumor and stromal compartments, and higher infiltration of NK cells, CD56^+^ NKT cells and CD68^+^ macrophages in the stromal compartment. *SMARCA4* mutated tumors had a significantly higher infiltration of CD163^+^ myeloid cells and CD163^+^CD68^+^ macrophages in the tumor compartment, and *APC* mutated tumors had a significantly higher infiltration of NKp46^+^ and CD56^+^NKp46^+^ NKT cells in the tumor and stromal compartments, and of CD68^+^ macrophages in the stromal compartment. Lastly, *ERBB3* and *SMAD4* mutated tumors had a significantly higher infiltration of mDCs in the tumor and stromal compartments, and *RNF43* mutated tumors had a significantly higher infiltration of iDCs in the tumor compartment. None of the investigated immune cell subsets differed by mutational status of *TP53* or *NF1*.

The abundance of different immune cells in relation to MMR status and morphology is shown in Additional File 4. The infiltration of several NKT cell populations and CD123^+^CD15^+^ granulocytes was significantly higher in MMR deficient than in MMR proficient I-type tumors, and the infiltration of CD163^+^ myeloid cells was significantly higher in MMR deficient than in MMR proficient PB-type tumors.

### Innate Immune Cell Density in Relation to Adaptive Immune Cell Density

Additional File 5 details the associations between innate immune cell infiltration and infiltration of several subpopulations of lymphocytes. Of note, high infiltration of NKp46^+^ NKT cells was significantly associated with high infiltration of single positive CD4 T cells, CD4^+^CD45RO^+^ T cells, CD4^+^ regulatory T cells, single positive CD8 T cells, CD8^+^CD45RO^+^ T cells, CD8^+^ regulatory T cells and B cells. High CD68^+^ macrophage infiltration was significantly associated with high infiltration of single positive CD8 T cells, CD8^+^CD45RO^+^ T cells and CD8^+^ regulatory T cells. Lastly, high infiltration of NKp46^+^CD56^+^ NKT cells was significantly associated with high infiltration of B cells.

### Prognostic Impact of Immune Cell Subpopulations

**FIGURE 4:**
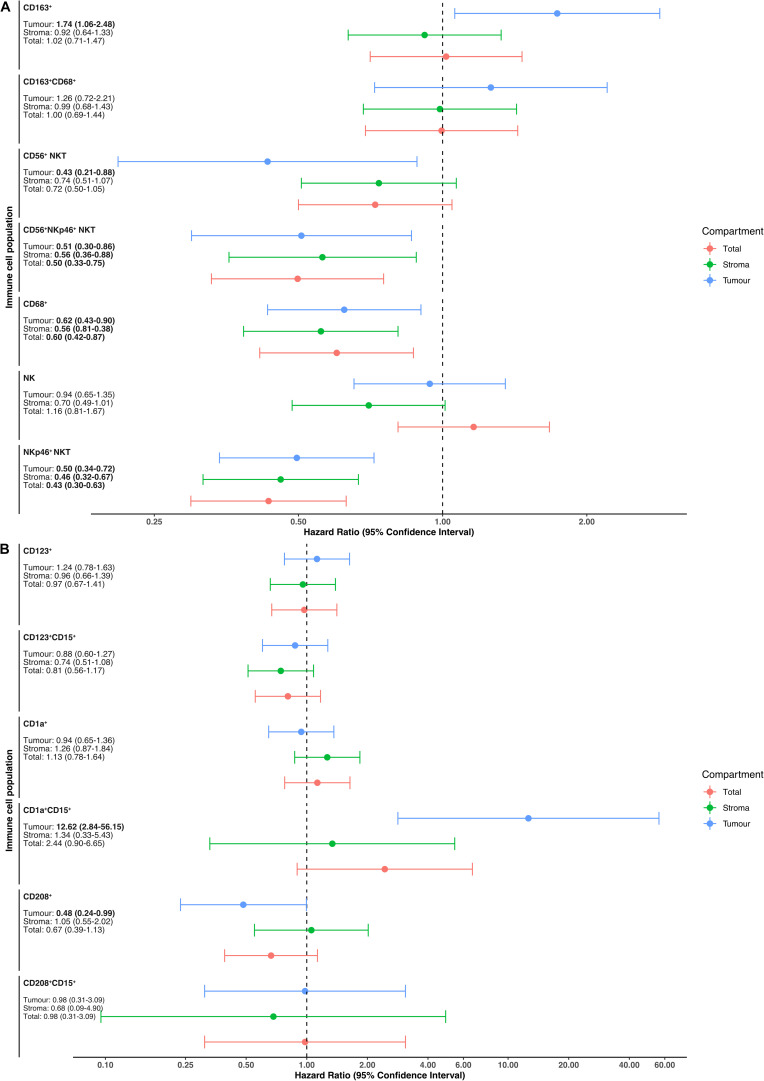
Prognostic impact of immune cell subsets in the entire cohort. **(A)** Forest plots depicting hazard ratios of death within 5 years according to the density of leukocytes characterized in panel 1. **(B)** Forest plots depicting hazard ratios of death within 5 years according to the density of leukocytes characterized in panel 2.

Hazard ratios for risk of death within 5 years in relation to the density of different immune cell subsets in the entire cohort are shown in Figure 4. A high total count of NKp46^+^ NKT cells (HR = 0.62, 95% CI 0.42–0.91) and NKp46^+^CD56^+^ NKT cells (HR = 0.63, 95% CI 0.41–0.98) was significantly associated with a prolonged OS in adjusted analysis, and high tumor nest (TN) infiltration of NKp46^+^ NKT cells (HR = 0.58, 95% CI 0.39–0.88) was also an independent factor of a prolonged OS. Furthermore, high TN infiltration of CD1a^+^CD15^+^ granulocytes (HR = 6.37, 95% CI 1.41–28.90) and CD123^+^CD15^+^ granulocytes (HR = 1.57, 95% CI 1.04–2.37) was significantly associated with a shorter OS in adjusted analysis. The stromal infiltration of immune cells did not confer any independent prognostic value *per se.*

Hazard ratios for risk of death within 5 years in relation to the density of different immune cell subsets in strata according to I-type and PB-type tumors are shown in Additional File 6. Neither the total count nor stromal immune cell infiltration *per se* were independent prognostic factors when stratifying for morphology. In I-type tumors, high TN infiltration of CD163^+^ myeloid cells was significantly associated with a shorter OS in adjusted analysis (HR = 3.11, 95% CI 1.02–9.48). In PB-type tumors, high TN infiltration of CD1a^+^CD15^+^ granulocytes (HR = 6.94, 95% CI 1.45–33.23) and CD123^+^CD15^+^ granulocytes (HR = 1.72, 95% CI 1.08–2.75) was significantly associated with a shorter OS, whereas high TN infiltration of NKp46^+^ NKT cells (HR = 0.57, 95% CI 0.36–0.93) was significantly associated with a prolonged OS in adjusted analysis.

### Prognostic Impact of Immunologically Hot and Immune-Excluded Phenotypes

In order to identify immunologically hot and immune-excluded tumors, respectively, the tumor to stromal infiltration ratio of different immune cells was analyzed. The rationale behind this was that enrichment of leukocytes in the tumor compartment could be an indicator of an efficient anti-tumor response, i.e., immunologically hot tumors, at least when it comes to effector immune cells. It was not possible to analyze the tumor to stroma ratio of mDCs, nor of CD208^+^CD15^+^ and CD1a^+^CD15^+^ granulocytes, due to the paucity of these cell populations. Hazard ratios for risk of death within 5 years according to the classification of cases into immunologically hot and immune-excluded tumors are shown in Additional File 7. Both in the entire cohort and in PB-type tumors, a high tumor to stroma ratio of iDCs (HR = 0.44, 95% CI 0.27–0.70 and HR = 0.51 95% CI 0.30–0.87, respectively) and NKp46^+^ NKT cells (HR = 0.65, 95% CI 0.44–0.96 and HR = 0.60, 95% CI 0.38–0.95, respectively) were independent factors of a prolonged OS. In I-type tumors, the classification into immunologically hot and immune-excluded tumors did not confer any prognostic value.

The distribution of immunologically hot and immune-excluded tumors did not differ significantly in relation to individual mutations or to MMR status (data not shown).

### Identification of Leukocyte Infiltration Signatures and Their Associations With Survival

**FIGURE 5:**
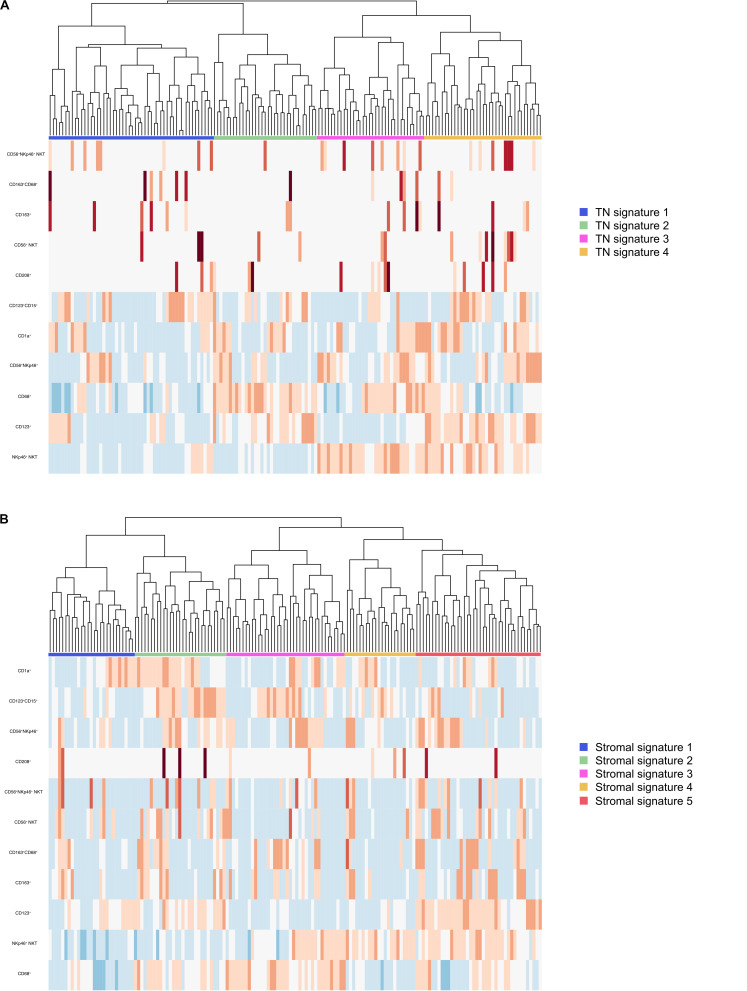
Identification of immune profiles in the entire cohort. **(A)** Heatmap illustrating unsupervised hierarchical clustering of patients by normalized infiltration densities of leukocytes in the tumor compartment. Blue color indicates low levels of infiltrating immune cells and red color indicates high levels of infiltrating immune cells. **(B)** Heatmap illustrating unsupervised hierarchical clustering of patients by normalized infiltration densities of leukocytes in the stromal compartment. Blue color indicates low levels of infiltrating immune cells and red color indicates high levels of infiltrating immune cells.

Regarding the total count, five immune signatures were identified using unsupervised hierarchal clustering, none of which were prognostic (data not shown). CD1a^+^CD15^+^ and CD208^+^CD15^+^ granulocytes were not included in the clustering due to the paucity of these cell populations. Cases were also clustered by leukocyte infiltration densities in the TN and stroma, respectively (Figure 5). Five distinct stromal immune infiltration signatures were identified, none of which were prognostic in adjusted analysis. Of the four identified TN signatures, none bore any prognostic significance, neither in the entire cohort nor in PB-type tumors, whereas in I-type tumors, TN signatures 1 and 3 were significantly associated with a shorter OS compared with TN signature 4 in adjusted analysis (HR = 4.71, 95% CI 1.29–17.20 and HR = 6.02, 95% CI 1.39–26.1, respectively). None of these immune signatures were significantly associated with individual mutations or MMR status (data not shown).

### Identification of Lymphocyte and Innate Immune Cell Infiltration Patterns and Their Associations With Survival

In order to identify more comprehensive patterns of immune cell infiltration, the most relevant immune cell populations described in the present paper were clustered together with the infiltration densities of previously described lymphocyte subpopulations (12) (Additional File 8). Cases were clustered into four distinct immune subtypes based on the total count. The four immune subtypes could be described as inflamed (high infiltration levels of all immune cells), myeloid inflamed – lymphocyte deprived (high infiltration levels of myeloid cells but low levels of lymphocytes), oasis (lower levels of most immune cells but high infiltration of NKT cells), and desert. In the entire cohort, myeloid inflamed – lymphocyte deprived, and desert subtypes were significantly associated with a shorter OS in adjusted analysis (HR = 2.30, 95% CI 1.30–4.07 and HR = 1.84, 95% CI 1.04–3.25, respectively) compared with the inflamed subtype. In PB-type tumors, the myeloid inflamed – lymphocyte deprived subtype was significantly associated with a shorter OS in adjusted analysis (HR = 2.33, 95% CI 1.16–4.68) compared with the inflamed subtype. In I-type tumors, none of the immune subtypes bore any prognostic significance. None of these immune signatures were significantly associated with individual mutations or MMR status (data not shown).

### The Impact of Leukocyte Spatiality and Cellular Interactions on Survival

Lastly, the average distance of each cancer cell to the nearest immune cell was calculated in order to estimate potential interactions. Hazard ratios for risk of death within 5 years in relation to the average distance of cancer cells to immune cells are shown in Additional File 9. In the entire cohort, a longer distance between cancer cells and CD56^+^ NKT cells (HR = 2.83, 95% CI 1.12–7.18) was significantly associated with a shorter OS in adjusted analysis. In I-type tumors, remoteness of cancer cells to CD68^+^ macrophages, pDCs and CD123^+^CD15^+^ granulocytes was significantly associated with a shorter OS in adjusted analysis (HR = 2.35, 95% CI 1.01–5.49, HR = 3.32, 95% CI 1.08–10.21 and HR = 5.29 95% CI 1.19–23.43, respectively), whereas in PB type tumors, remoteness of cancer cells to CD123^+^CD15^+^ granulocytes (HR = 0.30, 95% CI 0.14–0.64) was significantly associated with a prolonged OS in adjusted analysis.

**FIGURE 6:**
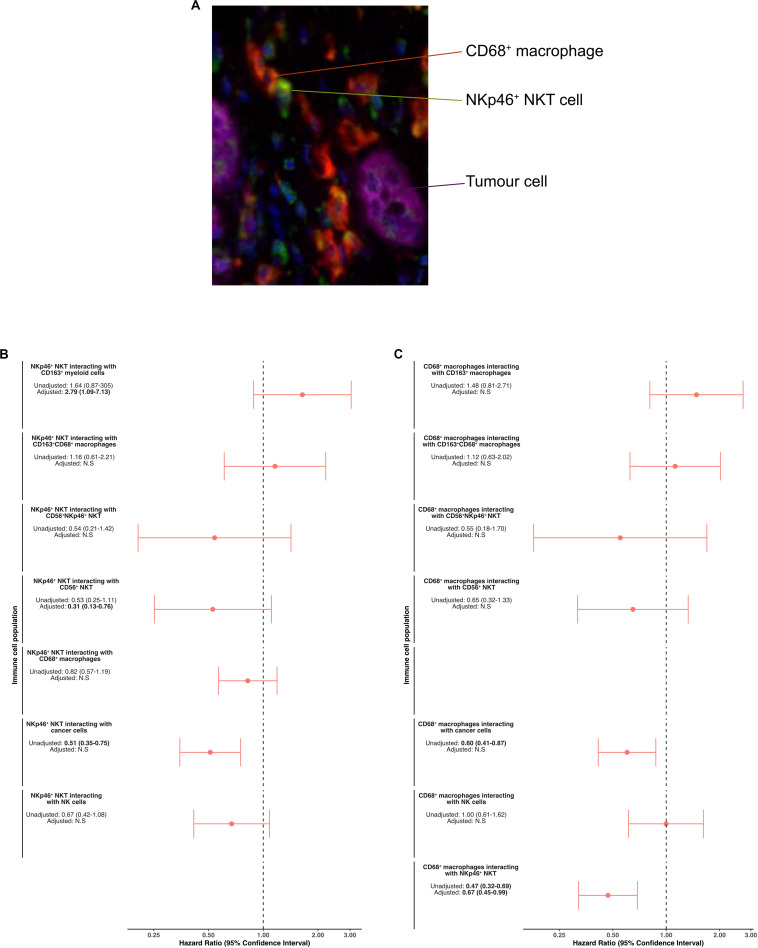
Cellular interactions and their impact on patient survival in the entire cohort. **(A)** Representative microphotograph of immunofluorescence staining illustrating NKp46^+^ NKT cells (green) within the interaction zone of a CD68^+^ macrophage (red). Cancer cells are visible as CK^+^ cells (purple). **(B)** Forest plots depicting hazard ratios of death within 5 years according to the interaction of NKp46^+^ NKT cells with other immune cells and cancer cells. **(C)** Forest plots depicting hazard ratios of death within 5 years according to the interaction of CD68^+^ macrophages with other immune cells and cancer cells.

Based on these results, we subsequently focused on NKT cells and CD68^+^ macrophages and their potential interactions with other immune cells and cancer cells, whereby a potential interaction event was defined as the presence of a cell within a 15 μm radius of a reference cell (Figure 6). In adjusted analysis, a high number of CD56^+^ NKT cells (HR = 0.56, 95% CI 0.37–0.84) in the interaction zone of NKp46^+^ NKT cells was significantly associated with a prolonged OS, whereas the opposite was seen for CD163^+^ macrophages (HR = 2.79 95% CI 1.09–7.13). Furthermore, a high number of NKp46^+^ NKT cells in the interaction zone of CD68^+^ macrophages was significantly associated with a prolonged OS in adjusted analysis (HR = 0.67 95% CI 0.45–0.99).

## Discussion

The immunological response to tumors is highly complex and the outcome of interactions between cancer cells and immune cells as well as the interactions between different immune cell classes depend upon a multitude of factors. The data presented in this paper are, to the best of our knowledge, the first to comprehensively describe the topography and phenotypic complexity of innate immune cell infiltration in periampullary adenocarcinoma, including pancreatic cancer, and to put it in a context of morphology and patient outcome.

The results demonstrate that high infiltration of several immune cell populations, especially NKT cells, was significantly associated with favorable clinicopathological factors, which is in line with previous findings regarding T and B lymphocytes (12).

Moreover, tumor morphology has been shown to be an important prognostic determinant in periampullary adenocarcinoma, more so than anatomical origin (1–4), and the herein presented results show that the quantity and quality of innate immune cell infiltration is dependent upon tumor morphology. Specifically, several immune cells, including macrophages and NKT cells, were significantly more abundant in I-type tumors than in PB-type tumors. In line with these findings, a previous paper using the same methodology demonstrated that T and B lymphocytes are also more abundant in I-type tumors (12). These findings are also supported by the considerably larger proportion of mismatch repair deficiency in I-type tumors (15), a feature that is often associated with a higher tumor mutational burden and tumor immunogenicity. Contrastingly, PB-type tumors may have properties that suppress immune cell infiltration and functionality.

The finding of a significantly lower infiltration of NKT cells in malignant tissue than in benign tissue is in line with a previous study on pancreatic cancer (21). NKT cells were also demonstrated to be less abundant in the tumor than in the stromal compartment. These results imply that cancer cells may have mechanisms to prevent NKT cell infiltration into tumors. Interestingly, we found that NKT cell, NK cell and CD68^+^ macrophage infiltration was lower in *KRAS* mutated than in *KRAS* wild-type tumors. This association was seen for both the tumor and the stromal compartments, but was more pronounced for the latter. NKT cells were more abundant in *APC* mutated tumors, which may well be explained by the fact that *APC* mutations were exclusively found in I-type tumors in the current cohort (4). The significantly lower infiltration of CD68^+^ macrophages in *CDKN2A* mutated tumors is in line with findings for other types of immune cells (12, 22), but may also merely be due to the fact that *CDKN2A* mutations are more common in PB-type tumors (4). Moreover, in the present study, mDC infiltration was found to be higher in *SMAD4* wt tumors, which is in line with a previous study using a different method to assess both immune cell infiltration and mutational status (23).

In I-type tumors, MMR deficiency was significantly associated with higher densities of NKp46^+^ NKT cells, CD68^+^ macrophages and CD123^+^CD15^+^ granulocytes, whereas in PB-type tumors, only the density of CD163^+^ macrophages differed by MMR status, which further emphasizes that tumors of this morphology are more immunologically cold (12, 24).

Notably, we managed to demonstrate the positive impact of NKT cell infiltration on survival by several metrics and not only by single phenotype characterization. A high tumor to stroma ratio of NKp46^+^ NKT cells was shown to be an independent prognostic factor, further supporting that patients with an active antitumor response by NKT cells fare significantly better.

Moreover, several prognostic immune cell signatures were identified by unsupervised hierarchal clustering of cases by immune cell infiltration densities. Interestingly, neither the total immune cell count nor the stromal infiltration was prognostic for any of the herein investigated immune cell signatures. However, TN immune signature 4, characterized by high densities of NKp46^+^ NKT cells, pDCs, NK cells and CD56^+^NKp46^+^ NKT cells, but low levels of CD68^+^ macrophages, was an independent predictor of a prolonged OS, but this association was only seen in I-type tumors. This observation may indicate that there is a difference between the two morphologies in what constitutes an efficient antitumoral immunological response. Additionally, the finding of the prognostic impact of defined immune signatures only being significant in a compartment specific context, further stresses the importance of taking topography into account when evaluating the clinical impact of immune cell infiltration into tumors.

Research into the role of NKT cells in pancreatic and periampullary adenocarcinoma has hitherto been sparse. We have previously shown that abundant CD56^+^ NK and NKT cell infiltration is associated with a prolonged survival using single marker immunohistochemical assessment (25). NKT cells, a CD1d restricted cell type, play an important role in immunosurveillance, however, their exact anti-tumor activity is not yet fully understood. Their function in tumor immunosurveillance is thought to be threefold, firstly by promoting antitumor response by other parts of the immune system, e.g., NK cells and CD8^+^ T cells, through IFNγ secretion, secondly by hindering macrophage tumor promotion, and thirdly by direct killing of malignant cells and other cells in the tumor microenvironment (26, 27). Therefore, NKT cells share characteristics of both conventional T cells and NK cells (27, 28).

Depletion of NKT cells in a pancreatic cancer model increased pancreatic intraepithelial neoplasia and disease progression (21), which may explain the relationship between NKT cell infiltration and a prolonged survival, seen in this and in a previous study (25). Several reports have shown that intratumoral NKT cells in some gastrointestinal cancers express lower amounts of cytokines, homing receptors, activation markers and proliferation markers than NKT cells in benign tissue (29–31), indicating cell anergy. A study on colorectal cancer showed that intratumoral NKT cells had higher levels of activation markers, such as FasL, than those derived from benign tissue (32). Future studies into the role and function of NKT cells in periampullary and pancreatic cancer is therefore warranted.

As for the populations of DCs, granulocytes and macrophages that were characterized in the present study, their infiltration levels were found to be significantly higher in the tumor than in the stromal compartment, and in malignant tissue than in paired benign tissue. Several of these populations were also demonstrated to be independent prognostic factors for shorter survival, including CD1a^+^CD15^+^ and CD123^+^CD15^+^ granulocytes in the entire cohort and CD163^+^ macrophages in both I-type and PB-type tumors. Previously, a study using single stain immunohistochemistry to assess infiltration of CD1a^+^ DCs and CD163^+^ macrophages in the same cohort demonstrated similar associations (33). Collectively, these findings are in line with previous studies showing that both tolerogenic immature DCs and tumor educated macrophages facilitate tumor progression in pancreatic cancer (34–36).

The current study revealed relatively low numbers of CD163^+^ myeloid cells and CD68^+^CD163^+^ macrophages compared to CD68^+^ macrophages. However, CD163^+^ cells have a tendency to accumulate in in necrotic regions and in intraglandular debris (37). In the present study, these regions were excluded from the analyses, thus likely providing a more reliable estimation of the infiltration levels of these cells. Moreover, the comparatively low CD163^+^ cell quantity is not likely due to methodological issues, since all slides were processed through the staining and imaging pipeline at the same time to avoid batch-effects, and there were no systematically low CD163^+^ numbers across samples.

When combining the densities of innate immune cell infiltration and lymphocyte infiltration we could identify four distinct patterns of immune cell infiltration; inflamed, myeloid inflamed – lymphocyte deprived, oasis and desert. These immune subtypes were significantly associated with survival in that the subtypes with the lowest levels of adaptive and innate lymphocytes, i.e., myeloid inflamed – lymphocyte deprived, and desert, had the shortest OS. This further highlights the detrimental effects of myeloid cells in the tumor microenvironment and the beneficial effects of lymphocytes. Interestingly, there were no significant differences in OS between the inflamed and oasis subtypes, the latter being characterized by all over lower infiltration levels of immune cells, with the exception of NKp46^+^ NKT cells. Furthermore, there were significant associations between infiltration of NKp46^+^ NKT cells and several phenotypic subpopulations of adaptive lymphocytes, including activated/memory cytotoxic T cells and activated/memory T helper cells and B cells. The association between adaptive lymphocytes and NKp46^+^ NKT cells observed in the current paper indicates that adaptive and innate lymphocytes act in concert in mounting an anti-tumor response. However, the oasis and inflamed immune subtypes (the former characterized by high levels of NKp46^+^ NKT cells but low levels of lymphocytes and the former by high levels of both) were associated with similar patient outcomes. This finding highlights that NKp46^+^ NKT cells inhabit a unique, beneficial niche in the tumor microenvironment.

When analyzing the spatial distribution on a single cell resolution, NKp46^+^ NKT cells and CD68^+^ macrophages were identified as being of particular importance when located in the direct vicinity of cancer cells. The impact of the spatial distribution of infiltrating immune cells differed by tumor morphology, but notably, in both tumor morphologies, a long distance between cancer cells and CD68^+^ macrophages as well as plasmacytoid DCs was associated with shorter OS. Additionally, by measuring the direct cellular interaction of CD68^+^ and CD163^+^ macrophages and NKp46^+^ NKT cells, we could demonstrate that the interaction of these immune cell populations are independent prognostic factors. Previously, it has been shown in a pancreatic cancer mouse model that NKT cells have a regulatory function on tumor infiltrating macrophages, indicating that NKT cells do not only have direct anti-tumor functions but also immune modulatory functions, especially on tumor associated macrophages (21). Further, in other solid tumors, NKT cells have been reported to colocalize to M2 polarized macrophages (27), leading to NKT mediated killing of macrophages (38, 39). Adding to this, a recent report showed that NKT cells are able to modulate macrophage function, promoting M1 over M2 polarization (31). Collectively, these data further support the hypothesis of a complex, but beneficial cross-talk between NKT cells and macrophages, and a favorable influence of NKT cells on an otherwise immunosuppressive tumor microenvironment.

## Conclusion

This study is the first to comprehensively describe the infiltration patterns of innate leukocytes in periampullary and pancreatic cancer. The results demonstrate that their spatial distribution, composition and prognostic impact differ by morphology. Additionally, the spatial mapping of single cells and their interactions provides a first *in situ* description of potential mechanisms of immunoregulation by the innate immune system in these cancers. Of particular interest is the noticeable clinical impact of NKp46^+^ NKT cells across all metrics, which should encourage further research into their potential role as orchestrators of important immunological events in the tumor microenvironment.

## Data Availability Statement

The raw data supporting the conclusions of this article will be made available by the authors, without undue reservation.

## Ethics Statement

The studies involving human participants were reviewed and approved by Ethics committee of Lund University (reference number 445/07). Written informed consent for participation was not required for this study in accordance with the national legislation and the institutional requirements.

## Author Contributions

SL, AM, KL, and KJ conceived and designed the experiments. SL and AM performed the experiments and analyzed the data. JE, MH, IH, BN, PM, and KJ contributed to reagents, materials, and analysis tools. SL wrote the manuscript. All authors read and approved the final manuscript.

## Conflict of Interest

The authors declare that the research was conducted in the absence of any commercial or financial relationships that could be construed as a potential conflict of interest.

## Abbreviations

DCs: dendritic cells
iDC: immature CD1a^+^ dendritic cells
mDC: mature CD208^+^ dendritic cells
MMR: mismatch repair
NK: natural killer cells
NKT: natural killer T cells
OS: 5-year overall survival
pDC: plasmacytoid CD123^+^ dendritic cells
TN: tumor nest
Treg: Regulatory T-cells.
