# Supplementary material for: Topographical Distribution and Spatial Interactions of Innate and Semi-Innate Immune Cells in Pancreatic and Other Periampullary Adenocarcinoma
Source: Front Immunol. 2020 Sep 10;11:558169. doi: 10.3389/fimmu.2020.558169 (PMC7511775; doi:10.3389/fimmu.2020.558169)

Additional File 6: Forest plots depicting hazard ratios of death within five years according to immune cell infiltration in A) panel 1 in I-type tumours B) panel 2 in I-type tumours C) panel 1 in PB-type tumours and D) panel 2 in PB-type tumours.

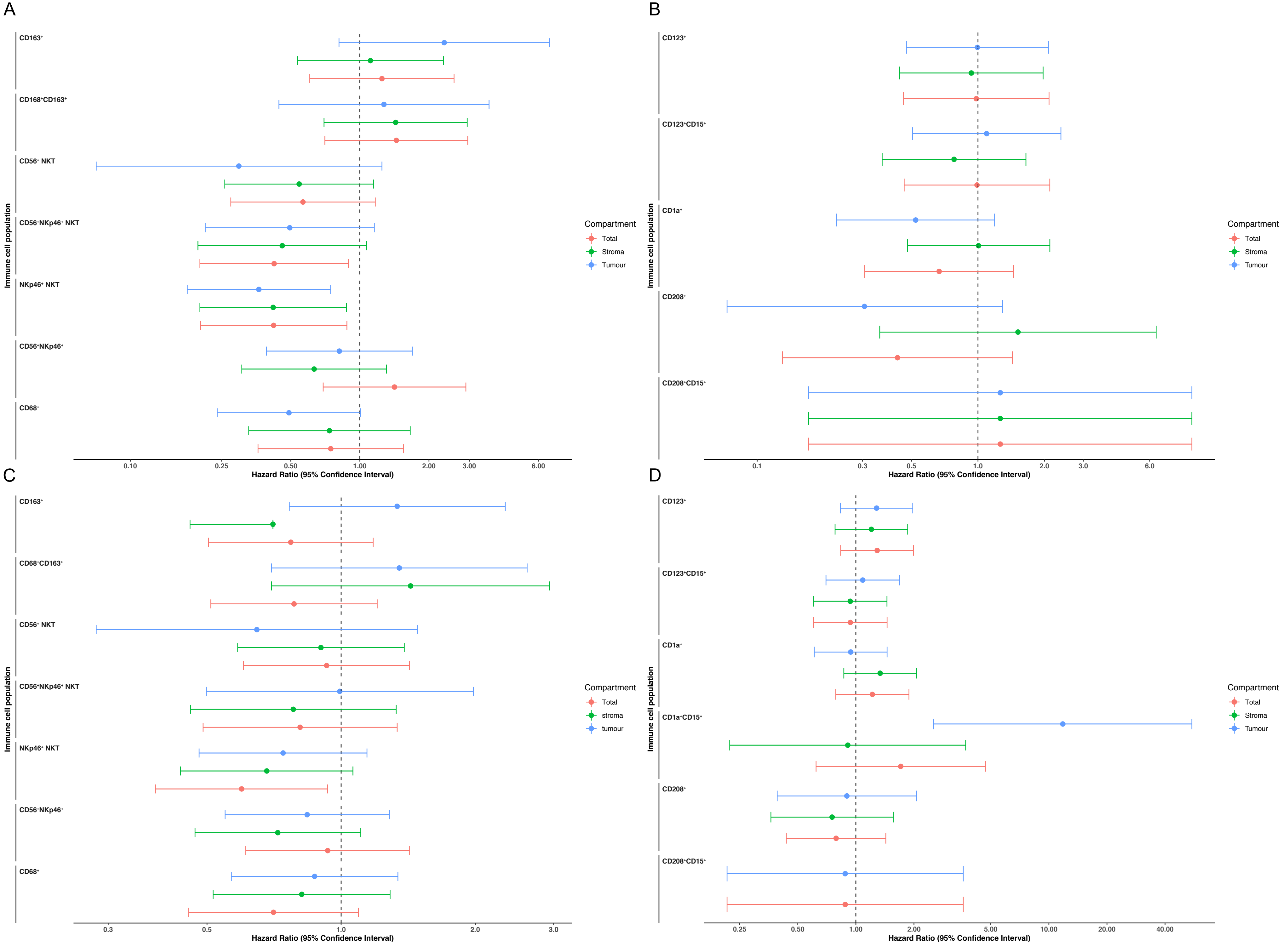

Supplement: Supplementary file 3 [file Image_3.pdf]
