# Supplementary material for: Topographical Distribution and Spatial Interactions of Innate and Semi-Innate Immune Cells in Pancreatic and Other Periampullary Adenocarcinoma
Source: Front Immunol. 2020 Sep 10;11:558169. doi: 10.3389/fimmu.2020.558169 (PMC7511775; doi:10.3389/fimmu.2020.558169)

Additional File 8: Identification of immune subtypes.

A) Clustering of cases by immune cell densities and identification of four immune subtypes. B) Median densities of immune cells stratified by immune subtype.

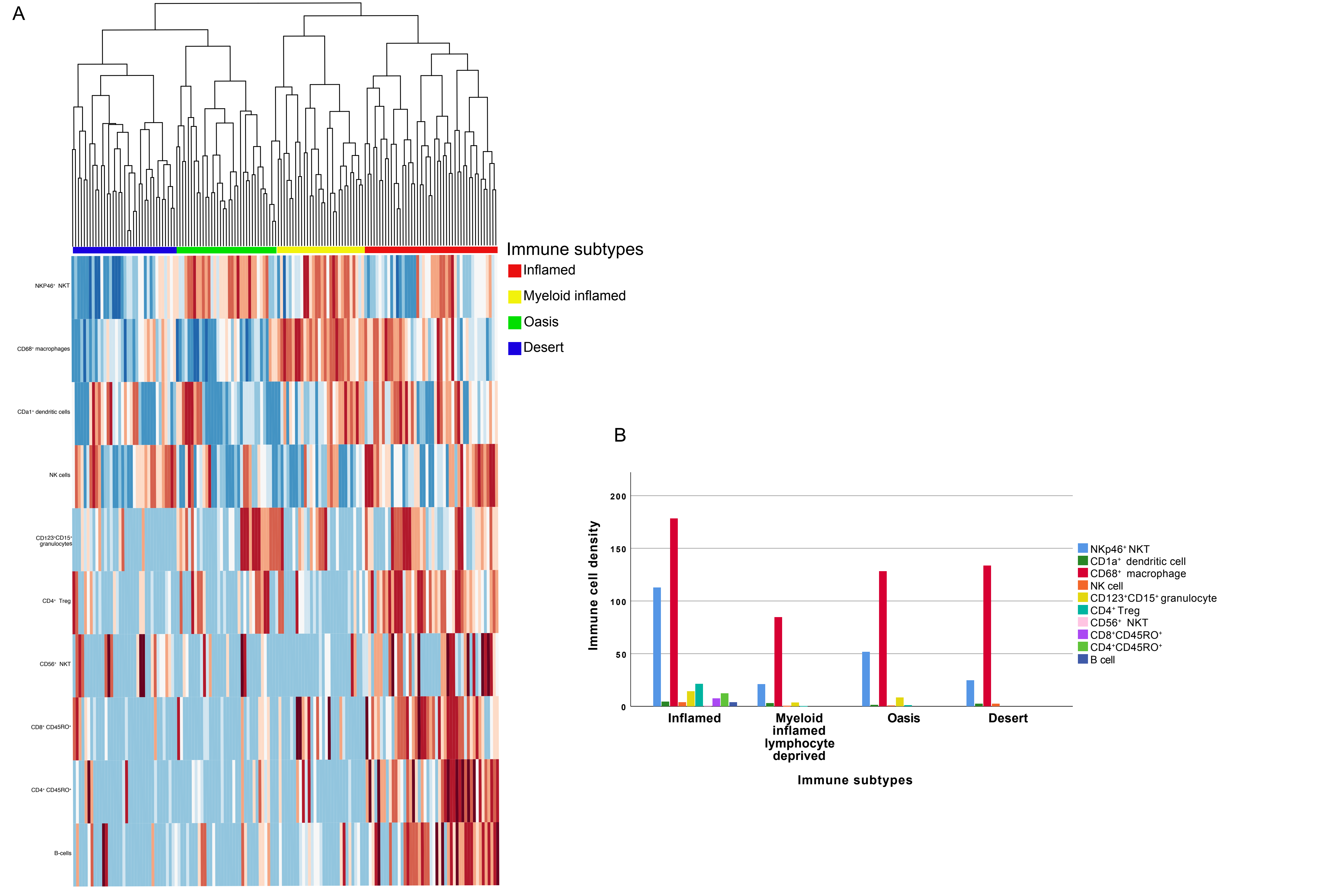

Supplement: Supplementary file 5 [file Image_5.pdf]
