# Supplementary material for: Topographical Distribution and Spatial Interactions of Innate and Semi-Innate Immune Cells in Pancreatic and Other Periampullary Adenocarcinoma
Source: Front Immunol. 2020 Sep 10;11:558169. doi: 10.3389/fimmu.2020.558169 (PMC7511775; doi:10.3389/fimmu.2020.558169)

Additional File 9: Forest plots depicting hazard ratios of death within five years according to average distance from immune cell subsets to cancer cells in A) panel 1 and B) panel 2.

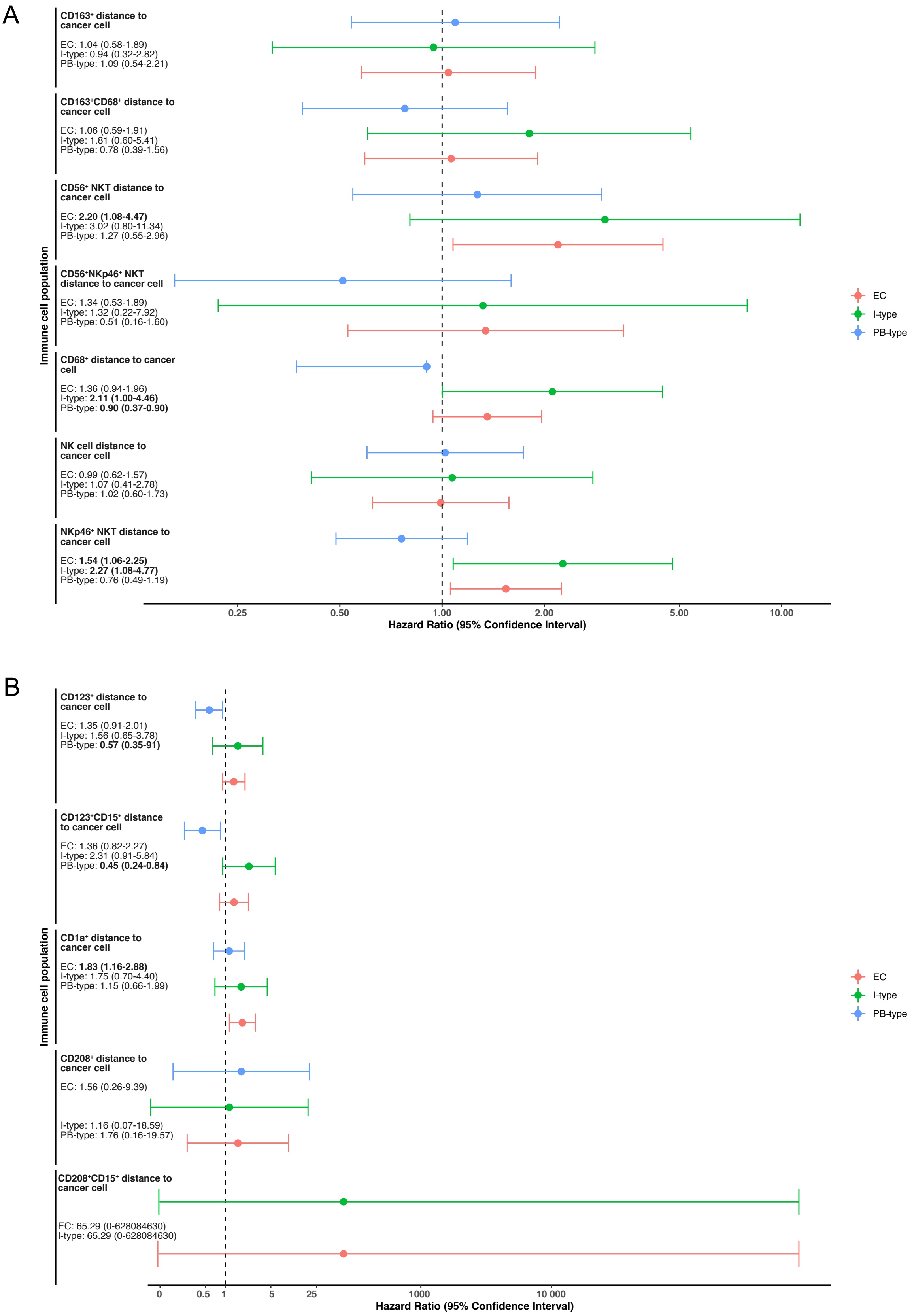

Supplement: Supplementary file 6 [file Image_6.pdf]
