# Supplementary material for: Topographical Distribution and Spatial Interactions of Innate and Semi-Innate Immune Cells in Pancreatic and Other Periampullary Adenocarcinoma
Source: Front Immunol. 2020 Sep 10;11:558169. doi: 10.3389/fimmu.2020.558169 (PMC7511775; doi:10.3389/fimmu.2020.558169)
Supplement: Supplementary file 7 [file Table_1.DOCX]

| Additional file 3. Differences in immune cell infiltration in tumor and stroma compartment in strata for common mutations (mean value). | | | | | | | | | | | | | |  |  |  |  |  |  |  |  |
| --- | --- | --- | --- | --- | --- | --- | --- | --- | --- | --- | --- | --- | --- | --- | --- | --- | --- | --- | --- | --- | --- |
|  | *KRAS* wt | *KRAS* mut | p-value | *RNF43* wt | *RNF43* mut | p-value | *SMAD4* wt | *SMAD4* mut | p-value | *SMARCA4* wt | *SMARCA4* mut | p-value | *CDKN2A* wt | *CDKN2A* mut | p-value | *APC* wt | *APC* mut | p-value | *ERBB3* wt | *ERBB3* mut | p-value |
| **Tumour compartment** |  |  |  |  |  |  |  |  |  |  |  |  |  |  |  |  |  |  |  |  |  |
|  |  |  |  |  |  |  |  |  |  |  |  |  |  |  |  |  |  |  |  |  | n.s |
| NK cells | n.s | n.s | n.s | n.s | n.s | n.s | n.s | n.s | n.s | n.s | n.s | n.s | n.s | n.s | n.s | n.s | n.s | n.s | n.s | n.s | n.s |
| CD56^+^ NKT | n.s | n.s | n.s | n.s | n.s | n.s | n.s | n.s | n.s | n.s | n.s | n.s | n.s | n.s | n.s | n.s | n.s | n.s | n.s | n.s | n.s |
| NKp46^+^ NKT | 35,654 | 6,02771 | 0.010 | 11,62395 | 38,23 | 0.046 | n.s | n.s | n.s | n.s | n.s | n.s | n.s | n.s | n.s | 11,026 | 78,133 | 0.001 | n.s | n.s | n.s |
| CD56^+^NKp46^+^ NKT | n.s | n.s | n.s | n.s | n.s | n.s | n.s | n.s | n.s | n.s | n.s | n.s | n.s | n.s | n.s | 0 | 1,0345 | <0.001 | n.s | n.s | n.s |
| CD68^+^ | n.s | n.s | n.s | n.s | n.s | n.s | n.s | n.s | n.s | n.s | n.s | n.s | 57,25299 | 36,339379 | 0.015 | n.s | n.s | n.s | n.s | n.s | n.s |
| CD163^+^ | n.s | n.s | n.s | 0 | 0 | 0.027 | n.s | n.s | n.s | 0 | 0,425 | <0.001 | n.s | n.s | n.s | n.s | n.s | n.s | n.s | n.s | n.s |
| CD163^+^CD68^+^ | n.s | n.s | n.s | n.s | n.s | n.s | n.s | n.s | n.s | 0 | 0 | 0.005 | n.s | n.s | n.s | n.s | n.s | n.s | n.s | n.s | n.s |
| CD1a^+^ | n.s | n.s | n.s | 1,558039 | 16,180691 | 0.048 | n.s | n.s | n.s | n.s | n.s | n.s | n.s | n.s | n.s | n.s | n.s | n.s | n.s | n.s | n.s |
| CD208^+^ | n.s | n.s | n.s | n.s | n.s | n.s | 0 | 0 | 0.019 | n.s | n.s | n.s | n.s | n.s | n.s | n.s | n.s | n.s | 0 | 0 | 0.002 |
| CD123^+^ | n.s | n.s | n.s | n.s | n.s | n.s | n.s | n.s | n.s | n.s | n.s | n.s | n.s | n.s | n.s | n.s | n.s | n.s | n.s | n.s | n.s |
| CD1a^+^CD15^+^ | n.s | n.s | n.s | n.s | n.s | n.s | n.s | n.s | n.s | n.s | n.s | n.s | n.s | n.s | n.s | n.s | n.s | n.s | n.s | n.s | n.s |
| CD208^+^CD15^+^ | n.s | n.s | n.s | n.s | n.s | n.s | n.s | n.s | n.s | n.s | n.s | n.s | n.s | n.s | n.s | n.s | n.s | n.s | n.s | n.s | n.s |
| CD123^+^CD15^+^ | n.s | n.s | n.s | n.s | n.s | n.s | n.s | n.s | n.s | n.s | n.s | n.s | n.s | n.s | n.s | n.s | n.s | n.s | n.s | n.s | n.s |
| **Stroma compartment** |  |  |  |  |  |  |  |  |  |  |  |  |  |  |  |  |  |  |  |  |  |
|  |  |  |  |  |  |  |  |  |  |  |  |  |  |  |  |  |  |  |  |  |  |
| NK cells | 1,2265 | 0 | 0.023 | n.s | n.s | n.s | n.s | n.s | n.s | n.s | n.s | n.s | n.s | n.s | n.s | n.s | n.s | n.s | n.s | n.s | n.s |
| CD56^+^ NKT | 1,0492 | 0 | 0.002 | n.s | n.s | n.s | n.s | n.s | n.s | n.s | n.s | n.s | n.s | n.s | n.s | n.s | n.s | n.s | n.s | n.s | n.s |
| NKp46^+^ NKT | 71,102 | 32,3004 | 0.045 | n.s | n.s | n.s | n.s | n.s | n.s | n.s | n.s | n.s | n.s | n.s | n.s | 46,179 | 182,3 | 0.012 | n.s | n.s | n.s |
| CD56^+^NKp46^+^ NKT | n.s |  |  | n.s | n.s | n.s | n.s | n.s | n.s | n.s | n.s | n.s | n.s | n.s | n.s | 0 | 0 | 0.036 | n.s | n.s | n.s |
| CD68^+^ | 261,48 | 168,981 | 0.017 | n.s | n.s | n.s | n.s | n.s | n.s | n.s | n.s | n.s | n.s | n.s | n.s | 202,99 | 371,9 | 0.009 | 212,3488 | 374,8625 | 0.025 |
| CD163^+^ | n.s | n.s | n.s | n.s | n.s | n.s | n.s | n.s | n.s | n.s | n.s | n.s | n.s | n.s | n.s | n.s | n.s | n.s | n.s | n.s | n.s |
| CD163^+^CD68^+^ | n.s | n.s | n.s | n.s | n.s | n.s | n.s | n.s | n.s | n.s | n.s | n.s | n.s | n.s | n.s | n.s | n.s | n.s | n.s | n.s | n.s |
| CD1a^+^ | n.s | n.s | n.s | n.s | n.s | n.s | n.s | n.s | n.s | n.s | n.s | n.s | n.s | n.s | n.s | n.s | n.s | n.s | n.s | n.s | n.s |
| CD208^+^ | n.s | n.s | n.s | n.s | n.s | n.s | n.s | n.s | n.s | n.s | n.s | n.s | n.s | n.s | n.s | n.s | n.s | n.s | n.s | n.s | n.s |
| CD123^+^ | n.s | n.s | n.s | n.s | n.s | n.s | n.s | n.s | n.s | n.s | n.s | n.s | n.s | n.s | n.s | n.s | n.s | n.s | n.s | n.s | n.s |
| CD1a^+^CD15^+^ | n.s | n.s | n.s | n.s | n.s | n.s | n.s | n.s | n.s | n.s | n.s | n.s | n.s | n.s | n.s | n.s | n.s | n.s | n.s | n.s | n.s |
| CD208^+^CD15^+^ | n.s | n.s | n.s | n.s | n.s | n.s | n.s | n.s | n.s | n.s | n.s | n.s | n.s | n.s | n.s | n.s | n.s | n.s | n.s | n.s | n.s |
| CD123^+^CD15^+^ | n.s | n.s | n.s | n.s | n.s | n.s | n.s | n.s | n.s | n.s | n.s | n.s | n.s | n.s | n.s | n.s | n.s | n.s | n.s | n.s | n.s |
|  |  |  |  |  |  |  |  |  |  |  |  |  |  |  |  |  |  |  |  |  |  |
