# Supplementary material for: Topographical Distribution and Spatial Interactions of Innate and Semi-Innate Immune Cells in Pancreatic and Other Periampullary Adenocarcinoma
Source: Front Immunol. 2020 Sep 10;11:558169. doi: 10.3389/fimmu.2020.558169 (PMC7511775; doi:10.3389/fimmu.2020.558169)
Supplement: Supplementary file 8 [file Table_2.DOCX]

| Additional file 4: Relationship between immune cell infiltration and mismatch repair status stratified for morphology. | | | | | | | | | | |
| --- | --- | --- | --- | --- | --- | --- | --- | --- | --- | --- |
|  | I-type |  |  | PB-type |  |  |  |  |  |  |
|  | pMMR | dMMR | p-value | pMMR | dMMR | p-value |  |  |  |  |
| **Tumour compartment** |  |  |  |  |  |  |  |  |  |  |
|  | n.s | n.s | n.s | n.s | n.s | n.s |  |  |  |  |
| NK cells | n.s | n.s | n.s | n.s | n.s | n.s |  |  |  |  |
| CD56^+^ NKT | n.s | n.s | n.s | n.s | n.s | n.s |  |  |  |  |
| NKp46^+^ NKT | 36,28 | 105,8 | 0.032 | n.s | n.s | n.s |  |  |  |  |
| CD56^+^NKp46^+^ NKT | n.s | n.s | n.s | n.s | n.s | n.s |  |  |  |  |
| CD68^+^ | 51,34 | 118,3 | 0.009 | n.s | n.s | n.s |  |  |  |  |
| CD163^+^ | n.s | n.s | n.s | n.s | n.s | n.s |  |  |  |  |
| CD163^+^CD68^+^ | n.s | n.s | n.s | 0 | 0 | 0.003 |  |  |  |  |
| CD1a^+^ | n.s | n.s | n.s | n.s | n.s | n.s |  |  |  |  |
| CD208^+^ | n.s | n.s | n.s | n.s | n.s | n.s |  |  |  |  |
| CD123^+^ | n.s | n.s | n.s | n.s | n.s | n.s |  |  |  |  |
| CD1a^+^CD15^+^ | n.s | n.s | n.s | n.s | n.s | n.s |  |  |  |  |
| CD208^+^CD15^+^ | n.s | n.s | n.s | n.s | n.s | n.s |  |  |  |  |
| CD123^+^CD15^+^ | 4,984 | 96,16 | 0.019 | n.s | n.s | n.s |  |  |  |  |
| **Stroma compartment** |  |  |  |  |  |  |  |  |  |  |
|  |  |  |  |  |  |  |  |  |  |  |
| NK cells | n.s | n.s | n.s | n.s | n.s | n.s |  |  |  |  |
| CD56^+^ NKT | n.s | n.s | n.s | n.s | n.s | n.s |  |  |  |  |
| NKp46^+^ NKT | n.s | n.s | n.s | n.s | n.s | n.s |  |  |  |  |
| CD56^+^NKp46^+^ NKT | n.s | n.s | n.s | n.s | n.s | n.s |  |  |  |  |
| CD68^+^ | 269,2 | 455,1 | 0.002 | n.s | n.s | n.s |  |  |  |  |
| CD163^+^ | n.s | n.s | n.s | 0 | 7,246 | 0.038 |  |  |  |  |
| CD163^+^CD68^+^ | n.s | n.s | n.s | 0 | 4,348 | 0.024 |  |  |  |  |
| CD1a^+^ | n.s | n.s | n.s | n.s | n.s | n.s |  |  |  |  |
| CD208^+^ | n.s | n.s | n.s | n.s | n.s | n.s |  |  |  |  |
| CD123^+^ | n.s | n.s | n.s | n.s | n.s | n.s |  |  |  |  |
| CD1a^+^CD15^+^ | n.s | n.s | n.s | n.s | n.s | n.s |  |  |  |  |
| CD208^+^CD15^+^ | n.s | n.s | n.s | n.s | n.s | n.s |  |  |  |  |
| CD123^+^CD15^+^ | 4,166 | 30,06 | 0.009 | n.s | n.s | n.s |  |  |  |  |
